# Comparative Analysis of Antimicrobial Antibodies between Mild and Severe COVID-19

**DOI:** 10.1128/spectrum.04690-22

**Published:** 2023-06-06

**Authors:** Ji Qiu, Anna Engelbrektson, Lusheng Song, Jin Park, Vel Murugan, Stacy Williams, Yunro Chung, Ericka Nelly Pompa-Mera, Jorge Luis Sandoval-Ramirez, Jose Antonio Mata-Marin, Jesus Gaytan-Martinez, Eliana Troiani, Maurizio Sanguinetti, Paola Roncada, Andrea Urbani, Giacomo Moretti, Javier Torres, Joshua LaBaer

**Affiliations:** a Center for Personalized Diagnostics, Biodesign Institute, Arizona State University, Tempe, Arizona, USA; b College of Health Solutions, Arizona State University, Phoenix, Arizona, USA; c Unidad de Investigación Médica en Enfermedades Infecciosas y Parasitarias, UMAE Hospital de Pediatría, Centro Médico Nacional Siglo XXI, Instituto Mexicano del Seguro Social, Mexico City, Mexico; d Hospital de Infectología, CMN “La Raza”, Instituto Mexicano del Seguro Social, Mexico City, Mexico; e Università Cattolica del Sacro Cuore, Rome, Italy; f Fondazione Policlinico Universitario A. Gemelli IRCCS, Rome, Italy; g Department of Health Sciences, University Magna Græcia of Catanzaro, Catanzaro, Italy; Thomas Jefferson University

**Keywords:** COVID-19, SARS-CoV-2, protein arrays, human coronavirus, herpesvirus, respiratory virus, virus antibody, antibody profiling

## Abstract

Patients with 2019 coronavirus disease (COVID-19) exhibit a broad spectrum of clinical presentations. A person’s antimicrobial antibody profile, as partially shaped by past infection or vaccination, can reflect the immune system health that is critical to control and resolve the infection. We performed an explorative immunoproteomics study using microbial protein arrays displaying 318 full-length antigens from 77 viruses and 3 bacteria. We compared antimicrobial antibody profiles between 135 patients with mild COVID-19 disease and 215 patients with severe disease in 3 independent cohorts from Mexico and Italy. Severe disease patients were older with higher prevalence of comorbidities. We confirmed that severe disease patients elicited a stronger anti-severe acute respiratory syndrome coronavirus 2 (SARS-CoV-2) response. We showed that antibodies against HCoV-229E and HcoV-NL63 but not against HcoV-HKU1 and HcoV-OC43 were also higher in those who had severe disease. We revealed that for a set of IgG and IgA antibodies targeting coronaviruses, herpesviruses, and other respiratory viruses, a subgroup of patients with the highest reactivity levels had a greater incidence of severe disease compared to those with mild disease across all three cohorts. On the contrary, fewer antibodies showed consistent greater prevalence in mild disease in all 3 cohorts.

**IMPORTANCE** The clinical presentations of COVID-19 range from asymptomatic to critical illness that may lead to intensive care or even death. The health of the immune system, as partially shaped by past infections or vaccinations, is critical to control and resolve the infection. Using an innovative protein array platform, we surveyed antibodies against hundreds of full-length microbial antigens from 80 different viruses and bacteria in COVID-19 patients from different geographic regions with mild or severe disease. We not only confirmed the association of severe COVID-19 disease with higher reactivity of antibody responses to SARS-CoV-2 but also uncovered known and novel associations with antibody responses against herpesviruses and other respiratory viruses. Our study represents a significant step forward in understanding the factors contributing to COVID-19 disease severity. We also demonstrate the power of comprehensive antimicrobial antibody profiling in deciphering risk factors for severe COVID-19. We anticipate that our approach will have broad applications in infectious diseases.

## INTRODUCTION

The pandemic of coronavirus disease 2019 (COVID-19) has exerted tremendous health, social, and economic burdens on humanity. COVID-19 is caused by severe acute respiratory syndrome coronavirus 2 (SARS-CoV-2), which belongs to the *Betacoronavirus* genus. In most patients, a healthy functional immune system will mount an effectual innate and adaptive immune response that is critical to control and resolve a SARS-CoV-2 infection (1). On the contrary, an impaired immune response will lead to either hypo- or hyperactivation of the immune system, resulting in excessive inflammation and even death (2). Many risk factors have been identified for the progression to severe COVID-19, including older age and underlying comorbidities (3). Deficiency in the production of type I interferons (IFNs) or the presence of autoantibodies against type I IFNs was shown to be associated with severe COVID-19 (4, 5). Many clinical parameters have been associated with the severity of COVID-19, including lymphopenia and increased concentrations of proinflammatory cytokines (6).

Antibodies play an important and complex role during SARS-CoV-2 infection (7). The magnitude and kinetics of antibody response to SARS-CoV-2 in COVID-19 has been extensively studied in different clinical settings (8). Anti-SARS-CoV-2 antibodies are important to facilitate diagnosis, assess population infection prevalence, and predict population immunity against SARS-CoV-2 infection. Furthermore, antibody classes, levels, and dynamics may reflect the level of initial viral load, duration of viral shedding, and the underlying innate and adaptive immune response to SARS-CoV-2 in COVID-19 patients, and they correlate with clinical characteristics (9). Previous studies showed that severe cases generally had an earlier IgM response and higher IgM and IgG levels against SARS-CoV-2 than did mild cases, indicating that humoral immunity to SARS-CoV-2 was stronger in severe cases than that in mild cases (10, 11). However, most antibody studies for COVID-19 have been restricted to the nucleocapsid (N) or the receptor binding domain (RBD) of the spike (S) proteins. Antimicrobial antibodies connote previous microbial infections. A comprehensive profiling of antibodies against human coronaviruses (HCoVs) and other important respiratory pathogens adds contextual information about patients’ infectious histories. This will improve our understanding of how prior infections and the resulting immunity affect the clinical course of COVID-19. Previous infections may also influence the risk of SARS-CoV-2 infection.

It is generally accepted that previous infections or vaccination of microorganisms can provide cross-protection of infections with related microbes whose antigens share similarities (12). However, even with microorganisms unrelated to an infection of interest, previous infections or vaccinations may train an individual’s immune system, with potential impact upon clinical trajectory, or even modifying risk of infection through a variety of mechanisms involving both the adaptive and innate immune responses (13). Antimicrobial antibodies produced in response to pathogen infection or vaccination can indicate previous exposure or vaccination. Furthermore, antibodies and antibody-producing B cells can also modulate the host innate and adaptive immune responses (14). Although there are no simple methods to assess a person’s immune fitness in response to infection, the levels of antibodies elicited by previous infections or vaccinations may serve as surrogates for an individual’s immune competence and predict the risk for developing severe disease.

Previous infections and antimicrobial antibody responses in COVID-19 patients have been exploited with limited scopes using targeted and/or peptide-based approaches (15–25). We performed a broad survey of antimicrobial antibodies against full-length microbial antigens in patients with severe or mild COVID-19 disease using protein arrays. Our microbial protein array platform can conveniently assess 2 different immunoglobulin types by using secondary antibodies conjugated to different fluorophores. In recognition of the importance of mucosal immunity in the infection of respiratory pathogens, we assayed the IgG and IgA antibodies against the antigens displayed on arrays. Our study focused on viruses, but we also included several antigens from 3 bacterial species relevant to human diseases. Helicobacter pylori infection is associated with the development of gastric cancer and many extragastric diseases. Antibodies against the flagellin of *Lachnospiraceae* are associated with inflammatory bowel disease. Mycobacterium tuberculosis infects the lung and causes tuberculosis. Mycobacterium tuberculosis also affects other parts of the body. We observed higher IgG and IgA anti-SARS-CoV-2 antibodies in severe relative to mild disease. We confirmed previous reports on upregulated antibody responses in severe disease. We also demonstrated that antibodies to a set of microbial antigens from additional herpesviruses and respiratory viruses were elevated in patients with severe disease. In contrast, very few antibodies showed higher reactivity in patients with mild disease.

## RESULTS

### Antibody profiling.

We assembled microbial antigen arrays comprising the NC proteins of all 7 human coronaviruses and 311 other full-length proteins selected from 77 different viruses and 3 bacteria (Table 1; see also Fig. S1 in the supplemental material), with the goal to survey the antimicrobial antibody landscape in COVID-19 patients. We profiled IgG and IgA antibodies against these microbial antigens in 3 independent sets of samples collected from patients with mild or severe COVID-19 disease in Mexico and Italy (Table 2; Table S1). Irrespective of the COVID-19 disease severity, many antigens elicited both IgG and IgA antibodies. However, IgG antibodies generally had higher reactivity than IgA antibodies against the same antigen. Ten antigens overlapped between the 20 antigens that elicited the strongest IgG antibody responses and the 20 antigens that showed the strongest IgA antibody responses (data not shown). The anti-Epstein-Barr virus BFRF2 had the highest IgG reactivity among the 318 antibodies profiled on the arrays. Interestingly, antibodies against several norovirus antigens showed the highest IgA reactivity.

**TABLE 1:** Microorganisms and number of antigens displayed on the microbial protein arrays

Microorganism name	No. of antigens
Aichi virus 1	1
Coxsackievirus A10	5
Coxsackievirus A16	1
Coxsackievirus A2	3
Coxsackievirus A22	6
Coxsackievirus A24	5
Coxsackievirus A4	3
Coxsackievirus A5	1
Coxsackievirus A6	5
Coxsackievirus A8	3
Coxsackievirus A9	5
Coxsackievirus B1	5
Coxsackievirus B2	1
Coxsackievirus B3	5
Coxsackievirus B4 (strain E2)	1
Coxsackievirus B5	6
Coxsackievirus B6	8
Echovirus 9	3
Echovirus E11	6
Echovirus E13	1
Echovirus E14	2
Echovirus E18	2
Echovirus E21	6
Echovirus E25	7
Echovirus E3	2
Echovirus E30	3
Echovirus E4	6
Echovirus E7	2
Enterovirus A71	3
Enterovirus C	8
Enterovirus D68	6
Helicobacter pylori	5
Hepatovirus A	1
Human coronavirus 229E	1
Human coronavirus HKU1	1
Human coronavirus NL63	1
Human coronavirus OC43	1
Human herpesvirus 1	17
Human herpesvirus 2	13
Human herpesvirus 3	10
Human herpesvirus 4	22
Human herpesvirus 5	16
Human herpesvirus 6A	2
Human herpesvirus 6B	3
Human herpesvirus 7	1
Human mastadenovirus A	4
Human mastadenovirus B	7
Human mastadenovirus C	13
Human mastadenovirus D	4
Human mastadenovirus F	10
Human metapneumovirus	4
Human orthopneumovirus	2
Human parainfluenza virus 1	1
Human parainfluenza virus 4a	1
Human parvovirus B19	3
Human respiratory syncytial virus B	2
Human respirovirus 3	1
Human rhinovirus A1	4
Human rotavirus C	3
Human rubulavirus 2	2
Influenza A virus [A/Puerto Rico/8/34/Mount Sinai (H1N1)]	3
Influenza A virus [A/Udorn/307/1972 (H3N2)]	4
Influenza B virus (B/Lee/1940)	1
*Lachnospiraceae*	1
Measles virus strain Schwarz	1
Measles virus strain Ichinose-B95a	1
MERS	1
Mumps virus	1
Mycobacterium tuberculosis	6
Norovirus GI	1
Norovirus GII	5
Parechovirus A	1
Rhinovirus B14	4
Rhinovirus C	5
Rotavirus strain wa	1
Salivirus NG-J1	1
SARS	1
SARS-CoV-2	1
Tioman virus	2
Vaccinia virus WR	1

**TABLE 2:** Patient cohorts and characteristics^*a*^

Characteristic	Cohort 1, Mexico		Cohort 2, Mexico		Cohort 3, Italy	
	Mild (*n* = 33)	Severe (*n* = 29)	Mild (*n* = 80)	Severe (*n* = 80)	Mild (*n* = 22)	Severe (*n* = 106)
% SpO_2_ (at admittance)	90.3 ± 1.2 [90.0, 95.0] (*n* = 33)	77.3 ± 11.5 [47.0, 91.0] (*n* = 29)			95.0 ± 4.47 [78, 100]	90.4 ± 6.0 [77, 100]
% SpO_2_ (lowest)					92.7 ± 4.8 [78, 100]	86.74 ± 4.93 [75, 92]
Age (yrs)	41.6 ± 10.5 [30.0, 77.0] (*n* = 32)	49.2 ± 11.4 [28.0, 75.0] (*n* = 29)	36.7 ± 9.5 [20.0, 59.0] (*n* = 77)	51.3 ± 16.7 [19.0, 89.0] (*n* = 78)	61.1 ± 16.7 [25, 90]	68.0 ± 10.2 [47, 88]
ALT (U/liter; normal range, 5–55)			24.9 ± 14.6 [8.0, 60.3] (*n* = 18)	48.8 ± 32.2 [10.0, 168.0] (*n* = 57)	73.3 ± 116.7 [7, 581]	50.0 ± 41.5 [5, 174]
AST (U/liter; normal range, 5–34)			23.1 ± 9.9 [12.7, 50.0] (*n* = 14)	36.0 ± 18.9 [11.0, 96.0] (*n* = 57)	45.2 ±27.3 [15, 107]	50.8 ± 36.2 [12, 121]
Bilirubin total					0.7 ± 0.3 [0.30, 1.60]	0.6 ± 0.2 [0.20, 0.90]
BMI			26.2 ± 4.0 [17.0, 41.1] (*n* = 77)	30.1 ± 5.4 [19.8, 42.4] (*n* = 55)		
C-reactive protein					48.4 ± 64.5 [0.5, 223.1]	94.5 ± 88.2 [0.5, 349.8]
Cholesterol (mg/dL)					154.2 ± 37.1 [79, 236]	143.8 ± 51.7 [57, 285]
Creatinine C			0.9 ± 0.1 [0.7, 1.4] (*n* = 23)	1.0 ± 1.4 [0.5, 11.0] (*n* = 57)	1.1 ± 1.3 [0.4, 9.1]	1.0 ± 0.7 [0.3, 3.5]
Days after the onset of symptoms and sample collection	33.2 ± 11.6 [15.0, 61.0] (*n* = 33)	12.7 ± 6.0 [1.0, 30.0] (*n* = 29)	35.4 ± 21.9 [0.0, 133.0] (*n* = 76)	14.8 ± 10.7 [1.0, 54.0] (*n* = 57)		
D-dimer (ng/mL)					1,566.8 ± 17,15.8 [190, 9,010]	3,944.1 ± 5,700.0 [190, 28,790]
Eosinophils (×1,000/μL)					0.1 ± 0.2 [0.0, 0.9]	0.2 ± 0.2 [0.0, 0.6]
Ferritin (mg/liter; normal range, 17.9–464]					717.9 ± 597.7 [50, 2,229]	1,058.1 ± 903.5 [55, 2,856]
Fibrinogen (mg/mL)					434.6 ± 170.7 [182,1001]	488.4 ± 178.4 [226, 963]
Gender	M = 16, F = 16 (*n* = 32)	M = 14, F = 15 (*n* = 29)	M = 40, F = 37 (*n* = 77)	M = 48, F = 31 (*n* = 79)	M = 15, F = 7	M = 74, F = 32
Glucose (mg/dL)			96.9 ± 12.7 [71.3, 126.0] (*n* = 23)	146.6 ± 83.7 [76.0, 477.0] (*n* = 57)	109.0 ± 30.9 [70, 187]	125.0 ± 60.4 [68, 310]
Hemoglobin (g/dL)			15.6 ± 2.4 [0.0, 18.8] (*n* = 77)	20.1 ± 29.7 [8.6, 205.0] (*n* = 57)	12.6 ± 1.6 [8.3, 17.4]	10.8 ± 2.1 [6.9, 14.3]
HDL-c (mg/dL)					32.3 ± 11.8 [19, 74]	28.7 ± 7.4 [11, 38]
LDH (U/liter)			236.7 ± 77.1 [142. 4,396.0] (*n* = 15)	376.4 ± 187.8 [152.0, 1037.0] (*n* = 56)	254.6 ± 96.3 [36, 486]	340.7 ± 112.4 [167, 623]
Leucocytes (×1,000/μL)			6.1 ± 1.8 [1.3, 12.6] (*n* = 77)	9.5 ± 4.2 [4.1, 25.3] (*n* = 57)	5.7 ± 2.4 [1.3, 12.5]	6.6 ± 4.1 [2.0, 17.8]
Lymphocyte count (×1,000/μL)			1.9 ± 0.6 [0.9, 4.3] (*n* = 77)	1.3 ± 1.6 [0.1, 10.6] (*n* = 57)	1.3 ± 0.7 [0.5, 3.8]	1.1 ± 0.9 [0.4, 2.8]
Monocytes					0.4 ± 0.1 [0.2, 0.8]	0.5 ± 0.3 [0.1,1.5]
Neutrophils (×1,000 μL)					3.8 ±2.1 [0.4, 9.6]	5.1 ± 4.1 [0.7, 17.0]
Obesity, overweight	6.1% (*n* = 33)	52.2% (*n* = 23)				
platelets (×1,000/μL)			269.4 ± 82.3 [140.0, 743.0] (*n* = 77)	293.4 ± 130.5 [92.0, 655.0] (*n* = 57)	298.5 ± 119.9 [113, 682]	274.8 ± 138.0 [91, 599]
Triglycerides (mg/dL)					164.3 ± 85.2 [72, 362]	174.9 ± 105.1 [69, 421]
Type 2 diabetes	3.0% (*n* = 33)	34.8% (*n* = 23)	0.0% (*n* = 77)	33.3% (*n* = 57)		
Urea (mg/dL)					34.0 ± 23.9 [0, 162]	49.0 ± 39.2 [0, 194]

a Data in each cell are shown in the following format: mean ± standard deviation [data range] (number of patients with data available for analysis). Shaded cells indicate results of comparison between mild and severe disease groups had significant *t* test *P* values of <0.05. Light gray indicates the group with a lower mean value, and dark gray shading indicates the group with a higher mean value for each paired comparison. Empty cells indicate no available data for patients with either mild disease or severe disease.

### Correlation of clinical features and measurements with disease severity.

All samples were collected in the early phase of the COVID-19 pandemic in Mexico and Italy. For simplicity and consistency between the study cohorts, patients with a positive PCR test who did not require hospitalization or supplemental oxygen were classified as having a mild disease, while those who were admitted to the hospital were classified as having a severe disease. We compared the clinical parameters between Mexican COVID-19 patients with mild and severe disease in each of the 2 cohorts separately and in combination (Table 2). Patients with severe disease had lower levels of saturation of peripheral oxygen (SpO_2_; 80% ± 12% in severe disease versus 90% ± 1% in mild disease; Wilcoxon rank-sum test, *P* < 0.001). Patients with mild disease were generally younger (38 ± 10 years in mild disease versus 51 ± 15 years in severe disease (Wilcoxon rank-sum test, *P* < 0.001) and had a lower body mass index (BMI; 26 ± 4 versus 30 ± 5 in severe disease; Wilcoxon rank-sum test, *P* < 0.001) and lower incidences of comorbidities such as type 2 diabetes mellitus (T2DM), overweight or obesity. Incidence of T2DM was higher in patients with severe disease: 33.8%, compared to 1% in mild cases (Fisher’s exact test, *P* < 0.001), which was corroborated by blood glucose concentrations (157 ± 87 mg/mL in severe versus 97 ± 13 mg/mL in mild cases; Wilcoxon rank-sum test, *P* < 0.001). Severe disease was also associated with higher overall leukocyte counts ([10.1 ± 4.8] × 10^3^/μL versus [6.1 ± 1.8] × 10^3^/μL in mild cases; Wilcoxon rank-sum test, *P* < 0.001) but lower lymphocyte counts ([1.2 ± 1.3] × 10^3^/μL versus [1.9 ± 0.6] × 10^3^/μL in mild cases; Wilcoxon rank-sum test, *P* < 0.001). Blood lactose dehydrogenase (LDH), alanine aminotransferase (ALT), and aspartate aminotransferase (AST) were also higher in severe disease.

Compared with the Mexican cohorts, the Italian cohort was older with a different gender distribution; however, the differences between severe and mild disease were generally consistent among the different cohorts for measurements available for comparison (Table 2). Severe patients in the Italian cohort were also significantly older and had lower levels of SpO_2_ and higher levels of LDH and C-reactive protein (CRP). The blood glucose concentrations and overall leukocyte counts were higher and the lymphocyte counts were lower in severe patients, although the differences did not reach statistical significance.

### Coronavirus antibodies.

**FIG 1:**
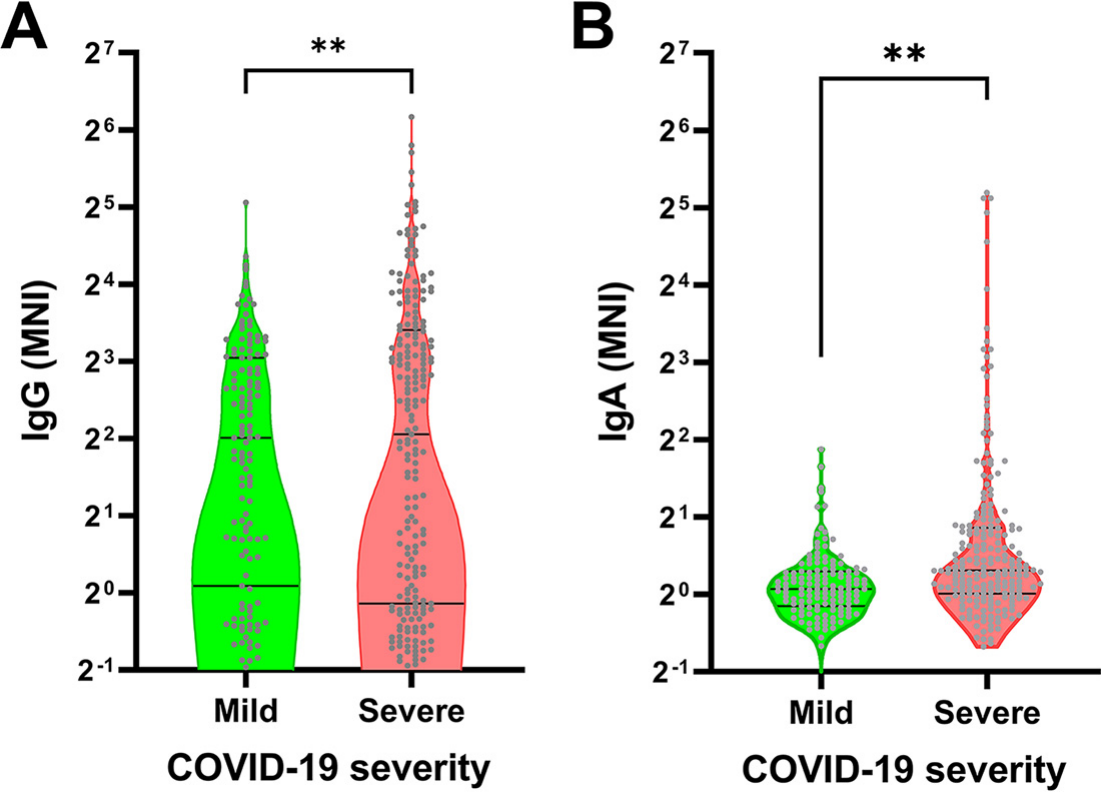
IgG (A) and IgA (B) antibodies against SARS-CoV-2 nucleocapsid protein (NC) were higher in severe COVID-19 disease than in mild disease. Each gray dot represents one COVID-19 patient. The lines indicate medians and upper and lower 25th percentiles. MNI, median normalized intensity. **, *P* < 0.01 (*t* test).

**FIG 2:**
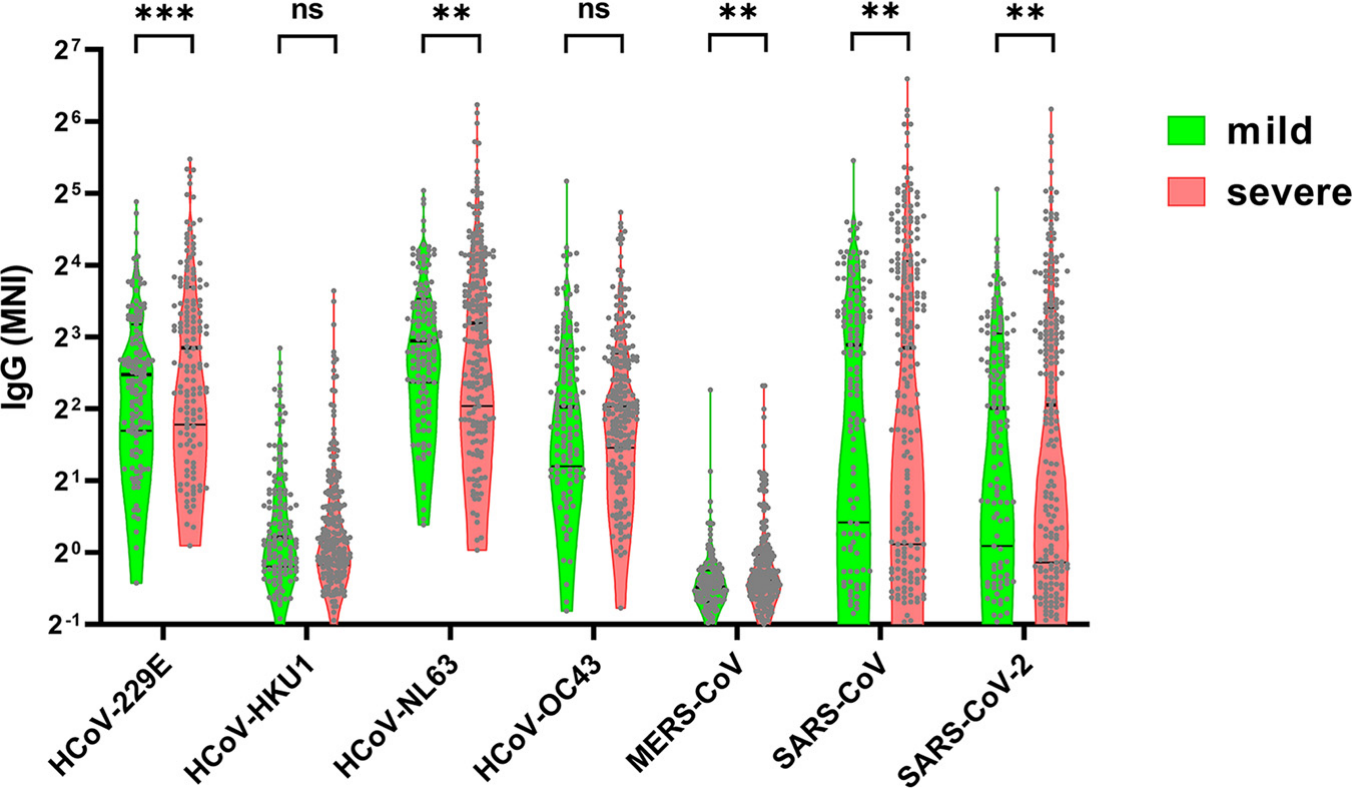
Comparison of IgG antibodies against nucleocapsid (NC) proteins from 7 human coronaviruses displayed on the microbial protein arrays in mild and severe COVID-19 disease. Abbreviations: HCoV-229, human coronavirus 229E; HCoV-HKU1, human coronavirus HKU1; HCoV-NL63, human coronavirus NL63; HCoV-OC43, human coronavirus OC43; MERS-CoV, Middle East respiratory syndrome-associated coronavirus; SARS-CoV, severe acute respiratory syndrome-associated coronavirus; SARS-CoV-2, severe acute respiratory syndrome-associated coronavirus 2. The lines indicate medians and upper and lower 25th percentiles. MNI, median normalized intensity. ***, *P *< 0.001; **, *P* < 0.01; ns, not significant (*t* test).

For patients in cohort 1, we also measured RBD antibodies using an Emergency Use Authorization (EUA) enzyme-linked immunosorbent assay (ELISA). Seropositivity as measured by the EUA ELISA and our microbial antigen arrays had >90% overall percent agreement (Fig. S2), although microbial antigen arrays showed a higher dynamic range to distinguish weak from strong antibody responses. Antibodies against the nucleocapsid protein of SARS-CoV-2 were significantly higher in patients with severe disease compared with those with mild disease (Fig. 1). We also assessed SARS-CoV-2 NC and RBD antibodies in 37 individuals who were exposed to COVID-19 patients but had no symptoms (Fig. S2), and over a quarter of the 37 were seropositive, which may suggest asymptomatic infection. NC antibodies for 6 human coronaviruses other than SARS-CoV-2 were also tested (Fig. 2). Nucleocapsid (NC) and spike (S) are the immunodominant coronavirus antigens. We studied antibodies against coronavirus NC proteins because of their elicitation only after natural infection and their robust detection on our microbial protein arrays (data not shown). Antibodies against the nucleocapsid proteins of SARS-CoV and the common cold coronaviruses HCoV-NL63 and HCoV-229E were also significantly higher in patients with severe disease compared to those with mild disease. However, antibodies against the nucleocapsid proteins of the common cold coronaviruses HCoV-HKU1 and HCoV-OC43 did not show significant differences between mild and severe disease (Fig. 2).

### IgG antibodies with higher reactivity in patients with severe disease.

**FIG 3:**
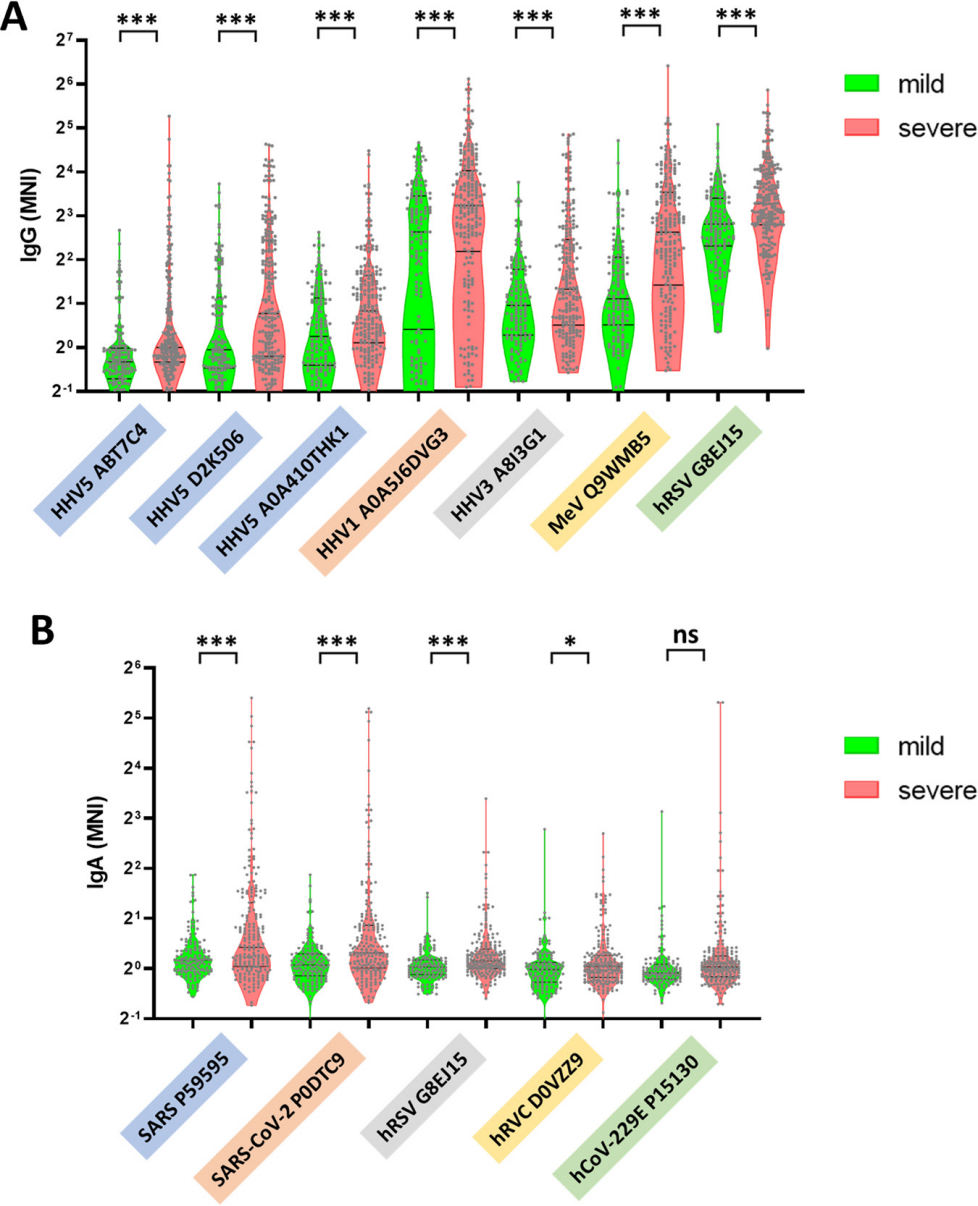
Selected IgG and IgA antibodies showing recurrent higher reactivity in severe COVID-19 disease. (A) The 7 most reactive IgG antibodies with higher reactivity in severe disease. These antibodies were selected from the 23 antibodies in Table 3. (B) The 5 IgA antibodies with high reactivity in severe disease. The *x* axis is labeled with the source virus name followed by the uniport ID of the target antigen. *x*-axis labels are colored based on microbial species. Abbreviations: HHV5, human herpesvirus 5 (cytomegalovirus); HHV1, human herpesvirus 1; HHV3, human herpesvirus 3 (varicella-zoster virus); MeV, measles morbillivirus; redundant hRSV, human respiratory syncytial virus (human orthopneumovirus); hRVC, human group C rotavirus; hCoV-229E, human coronavirus 229E; SARS-CoV, severe acute respiratory syndrome-associated coronavirus; SARS-CoV-2, severe acute respiratory syndrome-associated coronavirus 2. The lines indicate medians and upper and lower 25th percentiles. MNI, median normalized intensity. ***, *P* < 0.001; **, *P* < 0.01; *, *P* < 0.05; ns, not significant (*t* test).

We wished to examine whether there were specific antibody responses that were linked to the severity of disease outcome. We did not observe overall differences in microbial antibodies other than SARS-CoV-2 between mild and severe disease using the standard *t* test consistently across the 3 cohorts (data not shown). In recognition of the heterogeneity of COVID-19 and the heterogeneity of antibody responses when exposed to microbial antigens, we reasoned that the differences may be evident in a limited group of patients with higher levels of reactivity. Thus, we sought to rank the tightest linked responses by comparing the numbers of patients with mild or severe disease in the 10th decile, i.e., top 10% reactivity among all patients. For each antibody, among the patients with reactivity in the 10th decile, an odds ratio (OR) of >1 means that there were more patients with severe than mild disease, while an OR of <1 means more patients with mild disease than severe patients. This 10th decile OR analysis is similar to sensitivity at 90% specificity or partial area under the receiver operating characteristic (ROC)` curve (pAUC) analysis often used for biomarker research (26–30). To avoid overfitting, we kept the cohorts separately and looked for responses that were common to more than one. Antibody responses against 66, 83, and 71 of 318 microbial proteins demonstrated 10th decile odds ratios of >2 comparing severe and mild disease patients in cohort 1, 2, and 3, respectively (Fig. S3). Antibody responses against 36 of these 66 microbial proteins also showed 10th decile odds ratios of >2 in cohort 2. Antibody responses against 18 proteins showed 10th decile odds ratios of >2 in all 3 cohorts (Table 3, Fig. 3A; Fig. S3). Combining data for all samples, antibody responses against 63 proteins showed 10th decile odds ratios of >2 with *P* values of <0.05 (data not shown). The most reactive group of patients with the top 10% antibody responses against 6 of 16 human herpesvirus 5 (HHV5, also known as cytomegalovirus) antigens displayed on the arrays had higher reactivity in the severe disease patients in all 3 cohorts. We counted the number of HHV5 antigens that elicited antibody responses in each subject and found that the severe disease patients had antibody responses against higher numbers of HHV5 antigens than did mild disease patients (Fig. S4).

### IgG antibodies with higher reactivity in patients with mild disease.

We also analyzed antibodies with higher reactivity in mild disease and found that IgG antibody responses against 113, 57, and 85 microbial antigens demonstrated an OR of <0.5 in the 10th decile comparing severe and mild disease patients in cohorts 1, 2, and 3, respectively (Fig. S3). Antibody responses against 26 of these 113 microbial proteins also showed an OR of <0.5 in the 10th decile comparing severe and mild disease patients in cohort 2, while 9 of 26 showed an OR of <0.5 in the 10th decile comparing severe and mild disease patients in all 3 cohorts (Table S2, Fig. S3, Fig. S5).

### IgA antibodies with differential reactivity between mild and severe disease.

We analyzed differential IgA antibody production in mild and severe disease (Table 3 and Table S2) and found that IgA antibody responses against 5 microbial antigens had an OR of >2 in the 10th decile comparing severe and mild disease patients in cohorts 1, 2, and 3, with antibody responses against 4 of the 5 microbial antigens having *P* values of <0.05 (Table 3 and Fig. 3B). Three of the 5 microbial antigens were the NC proteins of HCoV-229E, SARS, and SARS-CoV-2 (Table 3). Three IgAs showed an OR of <0.5 in the 10th decile comparing severe and mild disease patients in the 3 cohorts, with 1 having a *P* value of <0.05 (Table S2).

**TABLE 3:** Target viral antigens and microorganisms for antibodies with high reactivity in severe COVID-19 disease^*a*^

Uniprot ID	Ig type	OR, severe vs mild					Fisher exact test *P* value, severe vs mild	*t* test *P* value, severe vs mild					Microorganism
		Cohort 1	Cohort 2	Cohort 3	Cohorts 1 and 2	Cohorts 1, 2, and 3	Cohorts 1, 2, and 3	Cohort 1	Cohort 2	Cohort 3	Cohorts 1 and 2	Cohorts 1, 2, and 3	
D2K4X3	IgG	8.3	3.4	6.5	4.3	4.2	1.6E−03	2.1E−02	8.1E−03	1.7E−01	8.9E−04	9.8E−04	Human herpesvirus 5
D2K552	IgG	2.5	8.3	2.7	4.3	4.2	1.6E−03	4.8E−02	1.6E−05	1.3E−01	3.6E−06	2.1E−05	Human herpesvirus 5
D2K4Z8	IgG	3.2	5	2.7	4.3	3.4	5.8E−03	7.5E−02	3.4E−04	2.7E−01	1.5E−04	7.0E−04	Human herpesvirus 5
D2K506	IgG	3.2	5	2.7	5.8	4.2	1.6E−03	1.1E−02	4.6E−06	1.1E−01	2.7E−07	3.4E−06	Human herpesvirus 5
A8T7C4	IgG	3.2	18.2	2.7	13.2	7.7	1.0E−04	4.2E−02	1.0E−03	1.4E−01	1.8E−04	1.3E−04	Human herpesvirus 5
A0A0G2TUZ2	IgG	3.2	5	2.7	4.3	3.4	5.8E−03	8.7E−02	5.3E−05	2.1E−01	2.8E−05	1.5E−04	Human herpesvirus 5
A8I3G1 (aa 1-525)	IgG	22.3	2.4	2.7	4.3	4.2	1.6E−03	3.6E−03	7.5E−03	5.4E−02	5.8E−04	1.1E−05	Varicella-zoster virus
A8I3G1 (aa 1-560)	IgG	8.3	2.4	2.7	2.6	3.4	5.8E−03	1.1E−02	1.1E−02	8.7E−02	1.1E−03	6.4E−05	Varicella-zoster virus
Q9WMB5	IgG	3.2	5	2.7	3.3	12.1	0.0E+00	9.4E−02	1.2E−04	1.1E−02	4.4E−05	3.2E−10	Measles strain Ichinose WT
A0A5J6DVG3	IgG	8.3	3.4	6.5	3.3	7.7	1.0E−04	6.4E−02	2.0E−03	2.8E−02	5.7E−04	1.6E−06	Human herpesvirus 1
J7FNP7	IgG	3.2	2.4	6.5	2.6	3.4	5.8E−03	3.1E−02	3.0E−02	2.2E−02	4.1E−03	1.2E−04	Human parainfluenza virus 1
P03466	IgG	8.3	3.4	6.5	2.6	2.3	4.6E−02	1.3E−01	1.5E−03	2.8E−03	7.8E−04	1.2E−05	Influenza A virus [A/Puerto Rico/8/34/Mount Sinai(H1N1)]
G9I248	IgG	3.2	4.1	4.4	4	3.6	6.6E−03	2.3E−01	1.9E−03	1.4E−01	6.5E−03	6.5E−03	Human alphaherpesvirus 2
G9I239	IgG	3.2	2.4	6.5	2.2	5.5	4.0E−04	1.7E−01	1.1E−02	3.7E−02	7.2E−03	5.7E−05	Human alphaherpesvirus 2
P06489	IgG	6.7	2.1	2.4	3.3	3.1	7.8E−02	3.6E−02	3.9E−02	7.0E−02	7.1E−03	2.7E−03	Human alphaherpesvirus 2
Q76UG7	IgG	2.4	2.1	2.2	2.2	2.4	1.20E−01	1.2E−02	2.0E−02	9.5E−02	3.5E−03	2.8E−04	Human alphaherpesvirus 2
G8EJ15	IgG	8.3	8.3	2.7	5.8	5.5	4.0E−04	5.0E−02	1.1E−03	1.7E−01	2.6E−04	5.5E−07	Human orthopneumovirus
P59595	IgG	22.3	8.3	2.7	28.3	12.1	0.0E+00	9.5E−04	1.2E−02	5.5E−01	4.2E−04	4.6E−03	SARS-CoV
G8EJ15	IgA	6.1	3.1	2.7	5.4	6.1	7.7E−03	3.6E−02	3.6E−03	1.4E−01	5.9E−04	3.9E−04	Human orthopneumovirus
D0VZZ9	IgA	3.2	4.2	2.2	3.3	3.2	4.4E−02	9.7E−02	1.2E−01	2.5E−01	5.4E−02	1.7E−02	Human rotavirus C
P15130	IgA	3.7	2.1	2.2	2.5	2.8	6.6E−02	6.4E−02	3.5E−01	2.1E−01	1.4E−01	5.8E−02	Human coronavirus 229E
P59595	IgA	22.3	41.2	2.7	61.7	12.1	0.0E+00	4.4E−03	2.0E−03	1.3E−01	6.5E−05	1.1E−04	SARS-CoV
P0DTC9	IgA	8.3	8.3	2.7	13.2	7.7	1.0E−04	1.1E−02	1.9E−03	1.5E−01	1.6E−04	6.4E−04	SARS-CoV-2

a In each cohort, for each antibody, the odds ratio between the number of patients with severe disease and the number with mild disease in the top 10% reactivity was calculated. Eighteen IgG antibodies and 5 IgA antibodies had odds ratios of ≥2 comparing patients with severe disease and those with mild disease in each of the 3 cohorts are listed. Also shown are odd ratios in the Mexican cohort (cohorts 1 and 2), odds ratios and associated Fisher exact test *P* values for the entire patient population (cohorts 1, 2, and 3), and *t* test *P* values for cohorts 1, 2, and 3 separately, cohorts 1 and 2 combined, and all 3 cohorts combined for these recurrent antibody responses against 23 microbial antigens.

### Correlation between antibody reactivity and clinical parameters.

**FIG 4:**
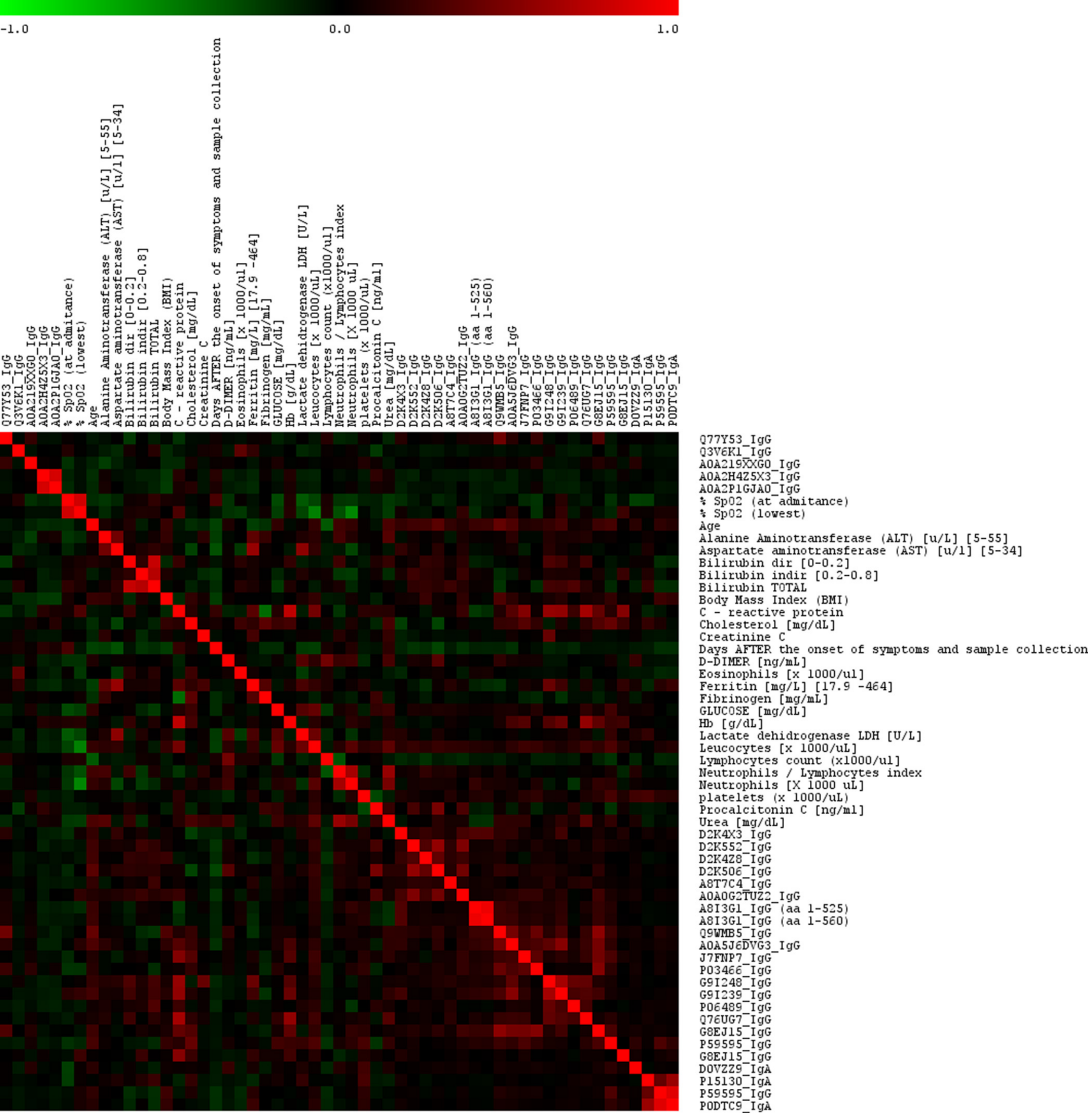
Correlations among clinical parameters and IgG and IgA antibodies with confirmed differential reactivity between mild and severe COVID-19 disease. Clinical parameters available for more than 10% of the COVID-19 patients were included in the analysis. Red indicates a positive correlation and green indicates a negative correlation.

We assessed the correlations among IgG and IgA antibodies with confirmed differential prevalence in mild and severe disease and clinical parameters (Fig. 4). Antibodies from the same or related viruses correlated positively. We observed correlations between antibody responses against antigens showing sequence homology, presumably due to shared epitopes, or antigens from the same virus, presumably due to shared presentation to the immune system. The levels of antibody with high reactivity in severe disease also correlated weakly with older age and with higher CRP, glucose, hemoglobin, and urea concentrations, all of which were commonly associated with severe disease.

## DISCUSSION

We performed an explorative study to compare viral antibodies in severe and mild COVID-19 disease and confirmed that antibodies against SARS-CoV-2 were higher in severe disease. Our study investigated three distinct cohorts of patients in Latin America and Europe. We discovered that patients in the subgroup with the highest levels of IgG and IgA antibodies against human herpesviruses and other respiratory viruses had a higher likelihood of severe COVID disease compared to those in the mild disease group, across all three cohorts. On the other hand, only a small number of antibodies consistently showed a higher occurrence in the mild illness group among the most responsive subgroup of patients across all three groups. These results are intriguing, though they require further study.

Many efforts have been put into the identification of clinical features and biochemical measurements that predict and/or are correlated with COVID-19 disease severity. Their elucidation is helpful in adopting appropriate preventive measures for different populations, managing COVID-19 patients effectively, and potentially minimizing disease mortality. There have been a few reports on the correlation of previous infections or vaccination with a reduced risk of infection or severe disease. Early in the pandemic, there were reports on the protection from BCG vaccine for tuberculosis in severe COVID-19, presumably due to trained innate immunity. However, other previous viral infections, such as hepatitis C virus (HCV), have been associated with poor outcome in COVID-19 patients, especially if accompanied by other risk factors, such as advanced age and hypertension (31).

More studies have focused on the effect of previous exposure and existing immunity to common cold coronaviruses on response to SARS-CoV-2, because antigens from different coronaviruses share high sequence homology and antibodies may cross-react among coronaviral antigens. For example, using samples from convalescent COVID-19 patients, Ladner et al. demonstrated that SARS-CoV-2 elicited antibodies that cross-recognized pandemic and endemic HCoV antigens at two Spike S2 subunit epitopes, and they postulated that previous HCoV exposures could influence the immune response to epitopes of SARS-CoV-2 (19). However, the possible protective role of immune response to common cold coronaviruses is still unclear. Our results showed similar reactivity patterns between SARS and SARS-CoV-2 NC antibodies, most likely due to cross-reactivity to highly homologous sequences. However, the fact that HCoV-229E and HCoV-NL63 NC but not HCoV-HKU1 or HCoV-OC43 NC antibodies had significantly higher reactivity in severe disease is intriguing, because HCoV-HKU1, HCoV-OC43, SARS, and SARS-CoV-2 all belong to the *Betacoronavirus* genus, whereas HCoV-229E and HCoV-NL63 belong to the *Alphacoronavirus* genus. These alphacoronavirus antibodies may be a marker for people with increased predisposition to severe infection. Alternatively, previous alphacoronavirus infections may train the innate immune system to overreact to SARS-CoV-2 infection.

Relatively few studies have assessed antibodies to other viruses in COVID-19 patients. Using a bacteriophage library displaying proteome-wide peptides from all human viruses, including coronaviruses, Shrock et al. found that COVID-19 patients who had required hospitalization exhibited higher seroprevalence rates for HHV5 and herpes simplex virus 1 (23), which is consistent with our results. There are several possible explanations for higher antibody reactivity against the viruses identified in our study. One possibility is the reactivation of previous infections with herpesviruses (18, 20, 22). One recent study demonstrated that reactivation of HSV-1 infection in the lower airway is highly associated with the worst prognosis and significantly increased COVID-19 mortality (21). Additionally, immunomodulatory and immunosuppressive therapies for COVID-19, while short-lived, at the high doses required in severe COVID-19 can also contribute to viral reactivation in these patients (17). Another possible explanation for the higher levels of antibodies against other respiratory viruses may be coinfection. Coinfection with other respiratory viruses, especially influenza virus, was associated with an increased risk for severe disease outcome and mortality in COVID-19 patients (15, 24, 25). Alternatively, high levels of antibodies to these respiratory viruses and vaccines, such as measles virus and rubella virus, suggest a hyperreactive immune system leads to severe disease (16).

Strengths of this study include the use of programmable protein arrays enabling the study of full-length antigens from many microorganisms and easy interpretation of antibody reactivity. On the other hand, studies that rely on random or viral peptides provide responses to localized folds or linear epitopes but cannot easily integrate such findings into a response to a specific protein. Bioinformatics analysis might be able to construct protein-level reactivity from peptide data, but that is rarely done, whereas full-length proteins also include conformational epitopes. The confirmation of antibody performance in 3 cohorts of different geographic regions speaks for the rigor of our findings. However, there are major limitations of this study. Samples prior to infection were not available, thus limiting our ability to distinguish association versus prediction. Antimicrobial antibody responses may be affected when, in the course of the SARS-CoV-2 infection, a patient is sampled. Samples from the Italian cohort and some from the 2 Mexican cohorts did not have information on the days between symptom onset and sample collection. For samples where this information was available, i.e., “Days between symptom onset and sample collection,” samples were generally collected earlier for hospitalized patients with severe disease than from outpatients with mild disease (Table 2). This raised the possibility that antibody levels varied due to time point in the disease course, which could be confounding. We compared the 23 recurrent differential antibodies (Table 3) between 55 patients with mild disease with samples collected ≤30 days after the onset of symptoms and 54 patients with mild disease with samples collected >30 days after the onset of symptoms. No antibodies showed significant Fisher exact *P* values for the 10th decile ORs (data not shown). This suggested that time during the course of the infection did not have a significant effect on antibodies to other organisms. However, future studies with more samples matched on the sample collection protocol are warranted to strengthen this conclusion.

In summary, our study demonstrated the power of an expansive microbiomics study of antimicrobial antibodies in understanding COVID-19 disease severity. Future studies with an expanded microbial antigen repertoire and a better-characterized patient cohort should improve our understanding of preexisting factors that may be a predictor or that are associated with disease severity. We believe an integrated approach to study patients’ serology, clinical characteristics, treatment, comorbidities, and demographic and behavior factors holds potential to advance the management of high-risk individuals and COVID-19 patients during this devastating pandemic.

## MATERIALS AND METHODS

### Patients and samples.

**(i) Mexican cohorts.** Subjects were informed about the nature of the study, and those willing to participate signed an informed consent letter prior to blood sample collection. Patients with confirmed COVID-19 were recruited from Hospital de Infectología, Centro Médico Nacional “La Raza” (IMSS; Mexico City, Mexico) from April 2020 to March 2021. Blood samples from both exposed subjects and hospitalized patients were collected in microtubes containing EDTA. All patients diagnosed with COVID-19 were confirmed by real-time PCR analysis of nasopharyngeal swab samples. None of the patients or exposed subjects had a history of anti-COVID-19 vaccination at the time of recruitment. The study was approved by the ethical committee of the National Research Council, Instituto Mexicano del Seguro Social, Mexico (protocol R-2020-785-082). Mild COVID-19 patients were defined as those who had a positive PCR test for SARS-CoV-2 and that did not require hospitalization or supplemental oxygen. Severe COVID-19 patients were defined as those who were hospitalized due to supplemental oxygen requirements. Nonsymptomatic health care workers were included in Mexican Cohort 1 and designated exposed individuals. Anthropometric and clinical characteristics (comorbidities, onset symptoms, oxygen saturation, disease severity), laboratory studies (complete blood test, glucose, lipids, ferritin, d-dimer, hepatic aminotransferases, bilirubin, urea, creatinine C, procalcitonin C), treatment (oxigenotherapy or mechanical ventilation, renal support, and antiviral, antimicrobial, or immunomodulatory therapies), and outcome data (i.e., duration of intensive care unit stay, mortality) were obtained from electronic medical records.

**(ii) Italian cohort.** Samples were collected from patients treated in Agostino Gemelli University Hospital (Rome, Italy) with the approval by the Hospital Ethical Committee (protocol N 0035744/20). All patients gave their consent to the storage of the sample in the Hospital Biobank and to the processing of their personal data (FPG PRO.1149). Whole-blood samples were collected using Greiner Bio-One Vacuette Tube Z Serum Sep Clot Activator tubes (Greiner bio-one, Kremsmünster, Austria) (FPG PRO.085). Blood samples were transported to the laboratory and centrifuged at 4,000 rpm for 5 min within 3 h of sample collection. Serum aliquots were stored at −80°C until shipment.

### Microbial protein array production and antimicrobial antibody profiling.

Our microbial protein arrays displayed 318 proteins from 3 bacteria and 77 viruses, including all 7 human coronaviruses, and other commensal and pathogenic microorganisms with an emphasis on those associated with respiratory tract infection (Table 1 and Fig. S1). Genes encoding microbial proteins cloned in pANT7_cGST were obtained from the DNASU plasmid repository (DNASU.org). This vector allows *in vitro* transcription from the T7 protomer coupled to protein expression using HeLa lysates on the protein arrays. Microbial protein arrays were produced using the Nucleic Acid Programmable Protein Array (NAPPA) technology (32, 33) by spotting plasmid DNA on silicon nanowell substrates as described elsewhere (34). Antimicrobial antibody profiling was also performed as previously reported (35). In brief, on the day of assay, C-terminal glutathione S-transferase (GST)-tagged microbial proteins were expressed using a HeLa cell lysate-based *in vitro* expression system and *in situ* capture of cospotted anti-GST antibody for their display on microbial protein arrays. Serum samples were applied on the microbial protein arrays followed by incubation with anti-human IgG and anti-human IgA antibodies labeled with different fluorophores. Arrays were scanned, and the images were analyzed by the Array-Pro image analysis software. Median fluorescence intensities at each spot were calculated for downstream data analysis.

Anti-RBD antibodies in a subset of samples were assayed using the SCoV-2 Detect IgG ELISA kit from InBios International, Inc. Measurement and interpretation of results were made according to the manufacturer’s instructions.

### Data analysis.

Spot intensities on each array were normalized by dividing the determined intensity by the median spot intensity of the corresponding array before statistical analysis to minimize the effects of the overall background differences among samples. Seropositivity was determined using the empirical median normalized intensity (MNI) cutoff of 2 as previously reported (28, 36). Descriptive statistics for demographic and clinical variables and normalized antibody reactivity were done using the SciPy python library (v1.4). The phylogeny tree was generated using Interactive Tree Of Life (iTOL v6). Heatmaps were generated using the Multiple Experiment Viewer (MeV 4.9.0), and dot plots were generated using GraphPad Prism v9.4.1. Venn diagrams were generated using the bioinformatics online tool at https://bioinformatics.psb.ugent.be/webtools/Venn/. Statistical tests such as Wilcoxon rank-sum or Fisher’s exact tests were performed using the R statistical software.

We observed the heterogeneity of microbial infections and antimicrobial immune responses in COVID-19 patients based on our exploratory data analysis. A standard approach, such as *t* test or Wilcoxon rank-sum test, was used to compare overall differences of antimicrobial antibody reactivities between two groups (patients with severe and mild disease). Our approach had limitations for analyzing our heterogeneous data and resulted in statistically nonsignificant results except for antibodies against SARS-CoV-2. For this reason, we used the 10th decile odds ratios, with the intention to capture the differences in the patient population with high antibody reactivity to microbial antigens.

To ensure the statistical rigor of our findings, we employed discovery and 2 independent confirmations using samples from 2 geographic regions to corroborate the discovery results to minimize false positives, as is commonly practiced in biomedical research. Similar approaches have been reported in publication by other groups and our own research (37–43).
