# Supplementary figures and images for: Comparative Analysis of Antimicrobial Antibodies between Mild and Severe COVID-19
Source: Microbiol Spectr. 2023 Jun 6;11(4):e04690-22. doi: 10.1128/spectrum.04690-22 (PMC10433851; doi:10.1128/spectrum.04690-22)

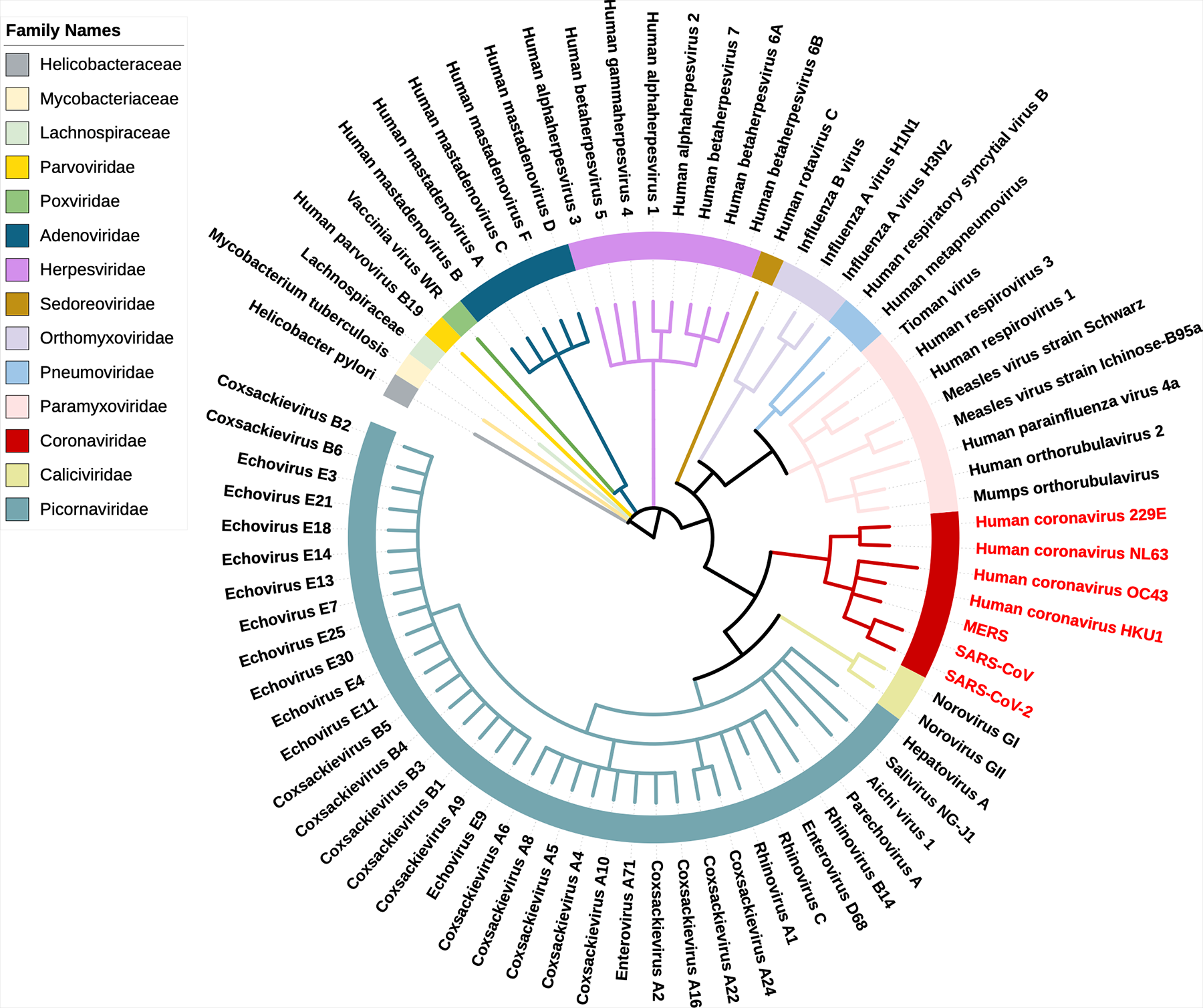

Supplement: Supplemental file 1 — Figure S1. Download spectrum.04690-22-s0001.tif, TIF file, 11.7 MB [file spectrum.04690-22-s0001.tif]

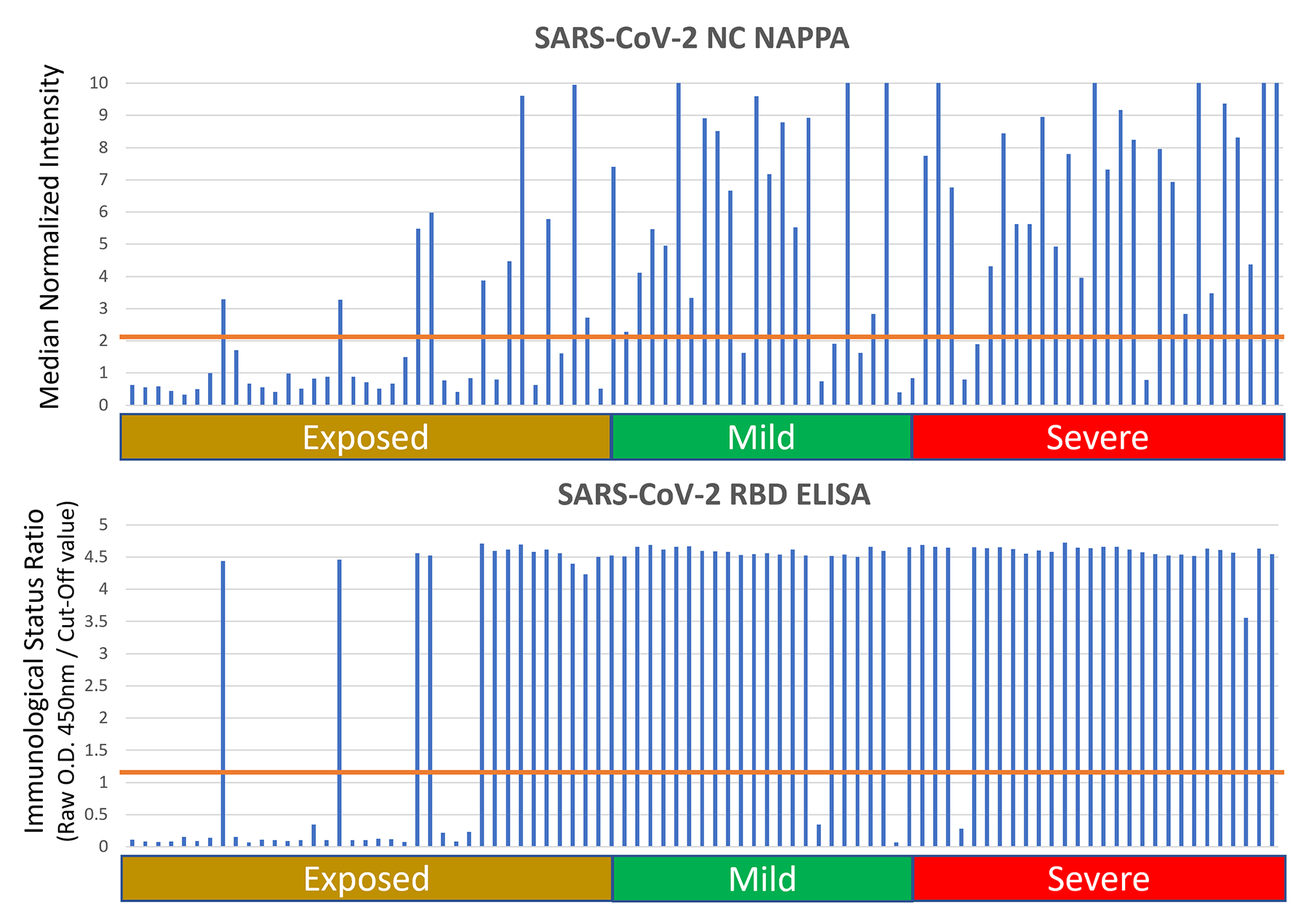

Supplement: Supplemental file 2 — Figure S2. Download spectrum.04690-22-s0002.tif, TIF file, 6.6 MB [file spectrum.04690-22-s0002.tif]

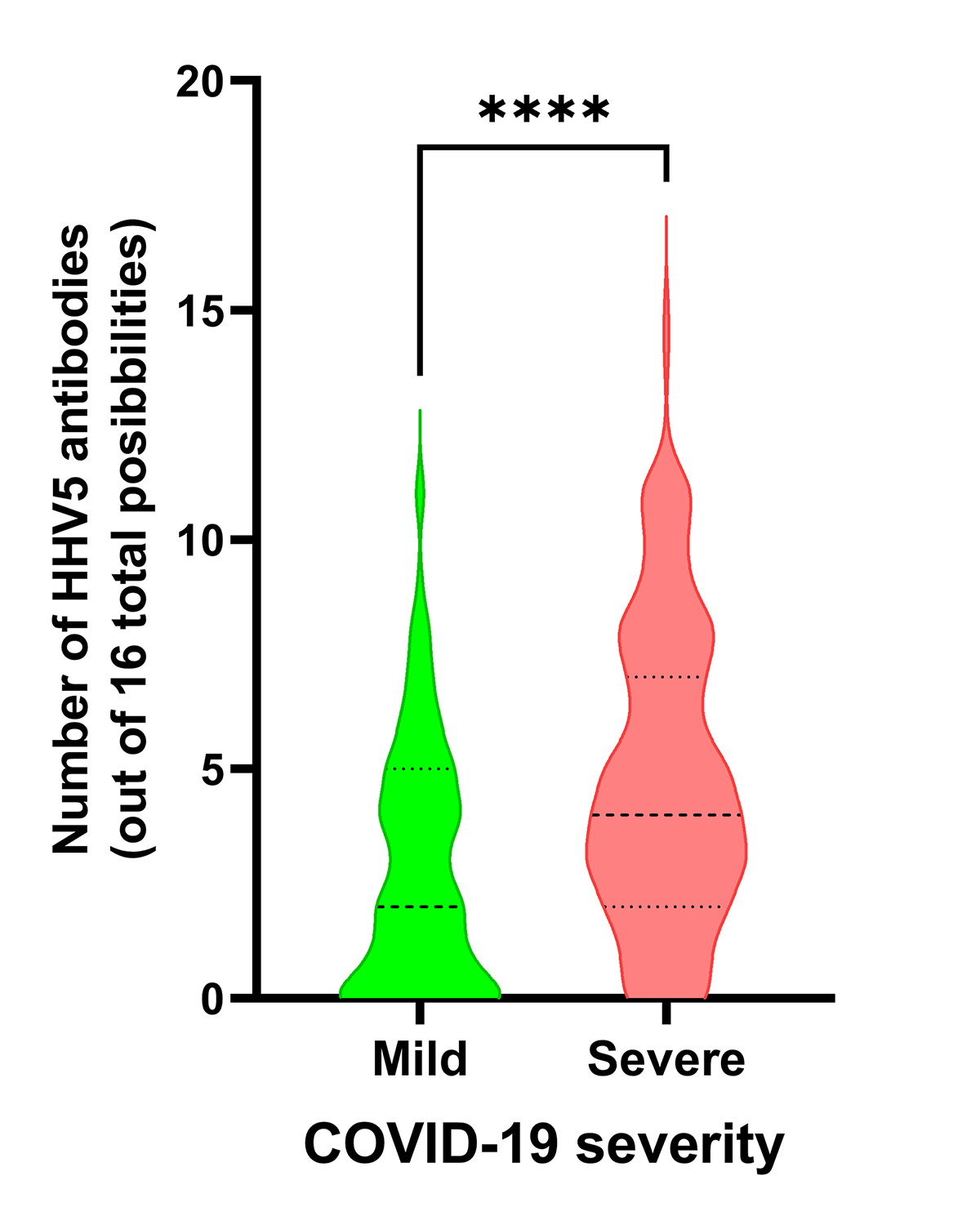

Supplement: Supplemental file 4 — Figure S4. Download spectrum.04690-22-s0003.tif, TIF file, 5.1 MB [file spectrum.04690-22-s0003.tif]

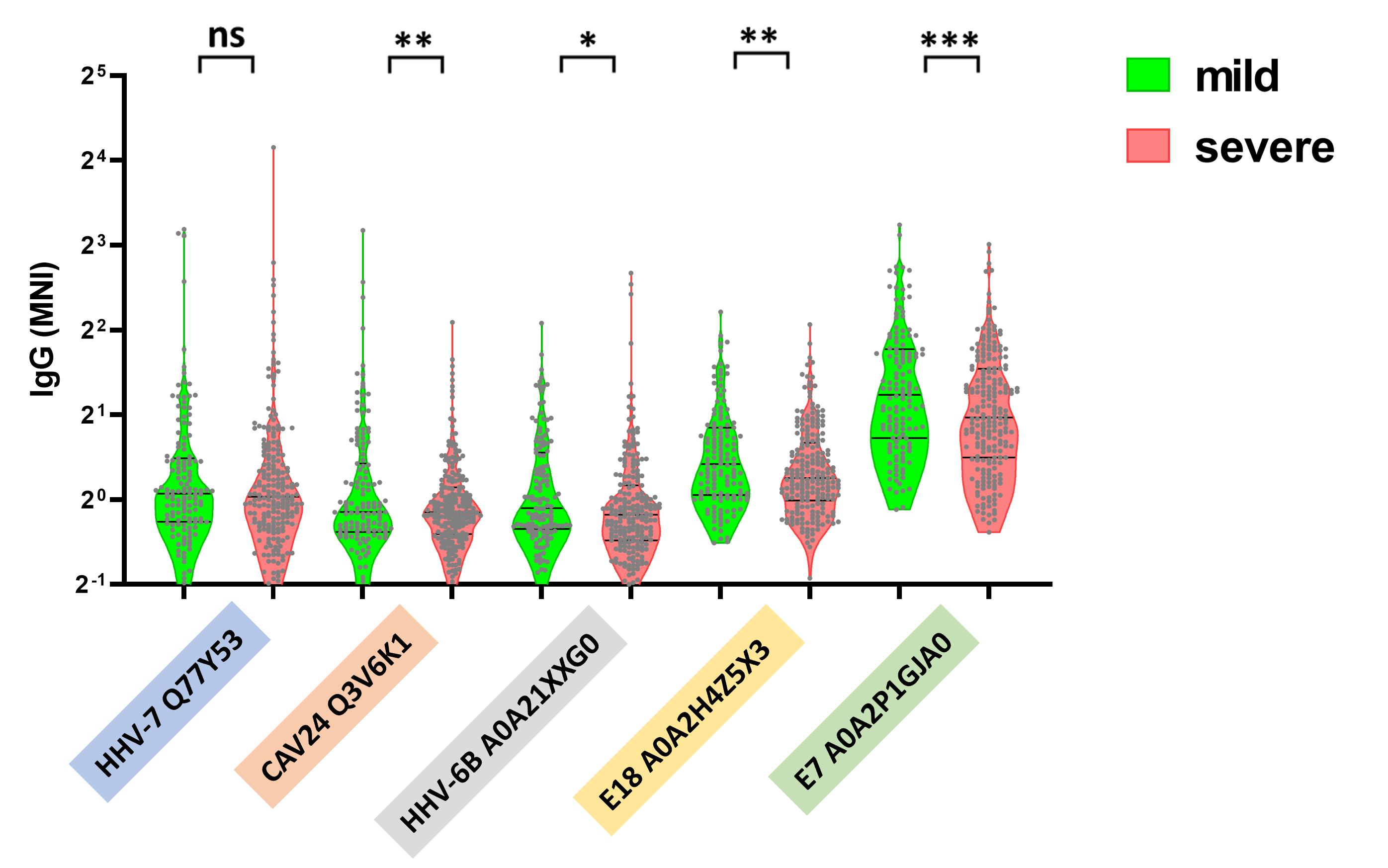

Supplement: Supplemental file 5 — Figure S5. Download spectrum.04690-22-s0004.tif, TIF file, 0.5 MB [file spectrum.04690-22-s0004.tif]

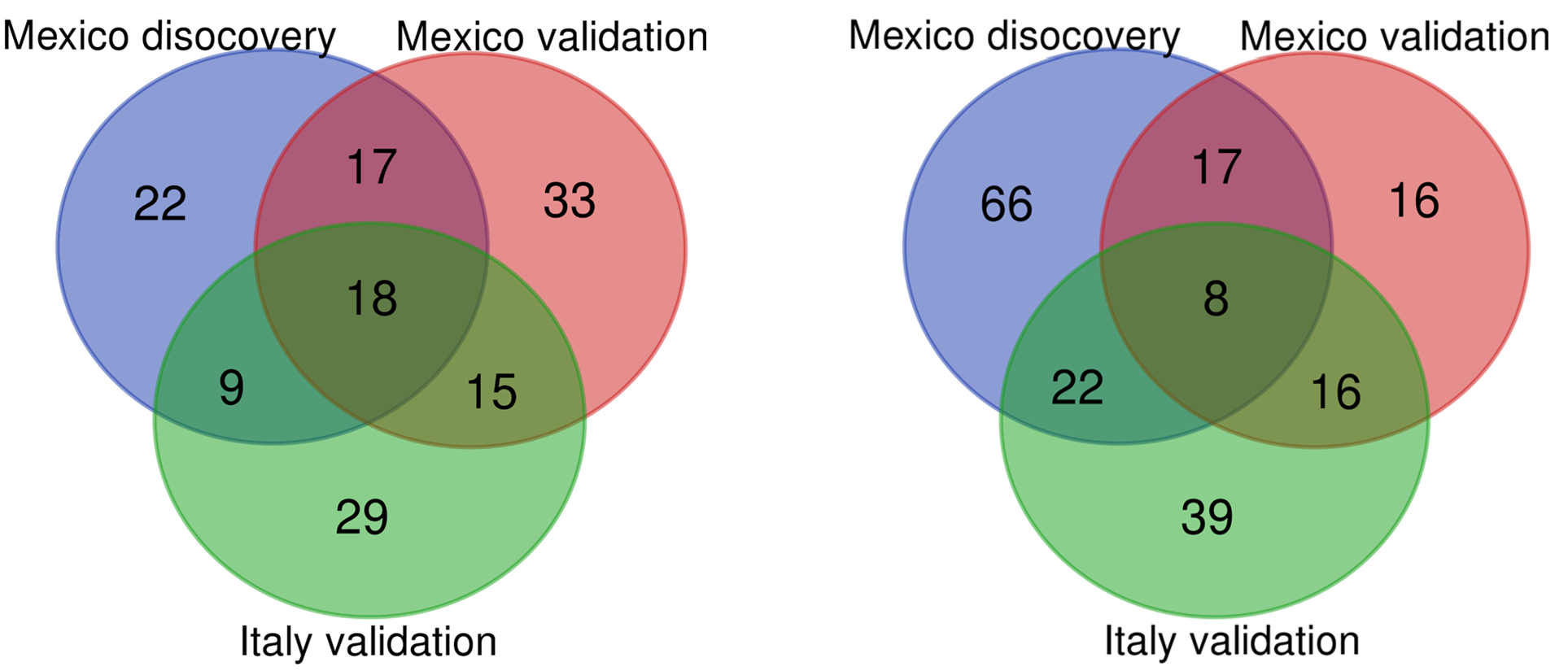

Supplement: Supplemental file 3 — Figure S3. Download spectrum.04690-22-s0005.tif, TIF file, 0.4 MB [file spectrum.04690-22-s0005.tif]
