# Supplementary material for: Comparative Analysis of Antimicrobial Antibodies between Mild and Severe COVID-19
Source: Microbiol Spectr. 2023 Jun 6;11(4):e04690-22. doi: 10.1128/spectrum.04690-22 (PMC10433851; doi:10.1128/spectrum.04690-22)
Supplement: Supplemental file 8 — Legends for all Tables S1 and S2 and Figures S1 to S5. Download spectrum.04690-22-s0007.docx, DOCX file, 0.03 MB [file spectrum.04690-22-s0007.docx]

**Table S1. IgG and IgA antibody responses to 318 microbial antigens.**

**Table S2. Antibodies with high reactivity in mild COVID-19 disease.** In each cohort, for each antibody, the odds ratio between the number of patients with severe disease and the number with mild disease in the top 10% reactivity was calculated. 9 IgG antibodies and 3 IgA antibodies had an odds ratio ≤ 0.5 comparing patients with severe disease and those with mild disease in each of the 3 cohorts are listed in this table. Also shown are odds ratios in the Mexican cohort (cohorts 1 and 2), odds ratios and associated Fisher exact test p-values for the entire patient population (cohorts 1, 2 and 3), and t-test p-values for cohort 1, 2, 3 seperately, cohorts 1 and 2 combined and all 3 cohorts combined for these recurrent antibody responses against 12 microbial antigens.

**Figure S1. Phylogenetic tree of microorganisms.** Coronaviruses are shown in red.

**Figure S2. Agreement between NAPPA and ELISA. Eight-nine (89) samples were assayed for NC antibodies by NAPPA (Top) and for RBD (Bottom) antibodies by SCoV-2 Detect™ IgG ELISA from Inbios internatioanl, Inc.** The orange line indicates the seropositivity cutoff of each assayThe positive percent agreement is ~90% and the negative percent agreement is 100%.

**Fig. S3.** Venn diagram showing overlaps of IgG antibodies with odd ratios of patients with severe disease to those with mild disease greater than 2 (left) or less than 0.5 (right) among the subgroups of patients with the top 10% antibody reactivity in 3 independent cohorts.

**Figure S4. Comparison between overall HHV5 response between mild and severe disease.** Sixteen HHV5 antibodies were assessed on the microbial protein arrays. We counted the number of HHV5 antibodies out of the 16 possibilities in each of the 350 COVID-19 patients and compared between the mild (n=135) and the severe (n=215) groups. Abbreviation: HHV5, Human herpesvirus 5. The lines indicate median, and upper and lower 25^th^ percentile.

**** t-test p-value < 0.0001

**Figure S5. Selected IgG antibodies showing recurrent higher reactivity in mild COVID-19 disease.** Antibody responses against 5 out the 12 microbial antigens in table S1 were selected. X-axis is labeled as the source virus name followed by the uniport ID of the target antigen. X-axis labels are colored based on microbial species. Abbreviations: HHV-7, Human betaherpesvirus 7; CAV24, Coxsackie virus A24; HHV-6B, Human betaherpesvirus 6B; E18, Echovirus E18; E7, Echovirus E7.

*** t-test p-value < 0.001

** t-test p-value < 0.01

* t-test p-value < 0.05

ns not significant
