# Supplementary material for: Comparative Analysis of Antimicrobial Antibodies between Mild and Severe COVID-19
Source: Microbiol Spectr. 2023 Jun 6;11(4):e04690-22. doi: 10.1128/spectrum.04690-22 (PMC10433851; doi:10.1128/spectrum.04690-22)
Supplement: Supplemental file 7 — Table S2. Download spectrum.04690-22-s0008.docx, DOCX file, 0.03 MB [file spectrum.04690-22-s0008.docx]

**Table S2. Antibodies with high reactivity in mild COVID-19 disease.**

| **Uniprot ID** | **Immunoglobulin Type** | **Odds ratio** between patients with severe disease and patients with mild disease in cohort 1 | **Odds ratio** between patients with severe disease and patients with mild disease in cohort 2 | **Odds ratio** between patients with severe disease and patients with mild disease in cohort 3 | **Odds ratio** between patients with severe disease and patients with mild disease in cohorts 1 and 2 | **Fisher exact test p-value** comparing patients with severe disease and patients with mild disease in cohorts 1, 2, and 3 | **T-test p-value** between patients with severe disease and patients with mild disease in cohort 1 | **T-test p-value** between patients with severe disease and patients with mild disease in cohort 2 | **T-test p-value** between patients with severe disease and patients with mild disease in cohort 3 | **T-test p-value** between patients with severe disease and patients with mild disease in cohorts 1 and 2 | **T-test p-value** between patients with severe disease and patients with mild disease in cohorts 1, 2, and 3 | **Microorganism** |
| --- | --- | --- | --- | --- | --- | --- | --- | --- | --- | --- | --- | --- |
| G9I254 | IgG | 0 | 0 | 0.2 | 0 | 1.5E-01 | 1.5E-01 | 4.0E-01 | 6.3E-01 | 2.6E-01 | 5.3E-01 | Human alphaherpesvirus 2 |
| Q77Y53 | IgG | 0.4 | 0.2 | 0.4 | 0.2 | 5.9E-01 | 3.2E-01 | 1.4E-03 | 7.6E-02 | 5.2E-03 | 4.1E-01 | Human herpesvirus 7 |
| Q3V6K1 | IgG | 0 | 0.2 | 0.4 | 0.1 | 5.6E-03 | 3.2E-02 | 3.8E-02 | 9.0E-02 | 7.7E-03 | 4.0E-03 | Coxsackievirus A24 |
| Q03053 | IgG | 0 | 0.5 | 0.4 | 0.3 | 4.4E-01 | 8.9E-02 | 2.4E-01 | 2.0E-02 | 9.1E-02 | 6.1E-02 | Coxsackievirus B5 |
| Q2XTY9 | IgG | 0 | 0.2 | 0.4 | 0.2 | 2.3E-01 | 7.8E-02 | 1.4E-01 | 6.8E-02 | 4.4E-02 | 3.9E-02 | Echovirus E11 |
| A0A219XXG0 | IgG | 0.3 | 0.4 | 0.5 | 0.4 | 6.8E-02 | 3.8E-01 | 7.5E-02 | 2.1E-01 | 8.8E-02 | 2.7E-02 | Human betaherpesvirus 6B |
| A0A7S5LIT7 | IgG | 0 | 0.5 | 0.4 | 0.4 | 1.4E-01 | 1.3E-01 | 1.2E-02 | 8.5E-02 | 6.9E-03 | 6.6E-04 | Coxsackievirus A8 |
| A0A2H4Z5X3 | IgG | 0.4 | 0.4 | 0.4 | 0.4 | 2.7E-02 | 9.8E-02 | 5.7E-02 | 5.1E-02 | 2.5E-02 | 3.1E-03 | Echovirus E18 |
| A0A2P1GJA0 | IgG | 0.4 | 0.4 | 0.4 | 0.5 | 2.7E-02 | 1.2E-01 | 6.2E-02 | 5.5E-02 | 3.4E-02 | 6.6E-04 | Echovirus E7 |
| T1UNE1 | IgA | 0 | 0 | 0.2 | 0 | 1.6E-01 | 5.8E-01 | 3.0E-01 | 1.3E-01 | 3.7E-01 | 2.7E-01 | Human mastadenovirus C |
| Q9YLM1 | IgA | 0 | 0 | 0.2 | 0 | 4.0E-04 | 2.9E-01 | 6.1E-04 | 2.0E-01 | 8.6E-04 | 5.7E-05 | Echovirus E4 |
| Q68T42 | IgA | 0 | 0 | 0.2 | 0 | 1.5E-01 | 1.9E-01 | 2.0E-01 | 5.2E-01 | 1.1E-01 | 1.0E-01 | Enterovirus D68 |
